# Facile synthesis of NiTe_2_-Co_2_Te_2_@rGO nanocomposite for high-performance hybrid supercapacitor

**DOI:** 10.1038/s41598-023-28581-5

**Published:** 2023-01-24

**Authors:** Maziar Farshadnia, Ali A. Ensafi, Kimia Zarean Mousaabadi, Behzad Rezaei, Muslum Demir

**Affiliations:** 1 Department of Chemistry, Isfahan University of Technology, Isfahan, 84156-83111 Iran; 2 Department of Chemistry & Biochemistry, University of Arkansas, Fayetteville, AR 72701 USA; 3 Department of Chemical Engineering, Osmaniye Korkut Ata University, Osmaniye, Turkey; 4 Tubitak Marmara Research Center, Material Institute, Gebze, 41470 Turkey

**Keywords:** Catalysis, Energy

## Abstract

The design of bimetallic tellurides that exhibit excellent electrochemical properties remains a huge challenge for high-performance supercapacitors. In the present study, tellurium is consolidated on CoNi_2_@rGO for the first time, to synthesize NiTe_2_-Co_2_Te_2_@rGO nanocomposite by using a facile hydrothermal method. As-prepared NiTe_2_-Co_2_Te_2_@rGO nanocomposite was characterized by EDS, TEM, FESEM, Raman, BET, XRD, and XPS techniques to prove the structural transformation. Upon the electrochemical characterization, NiTe_2_-Co_2_Te_2_@rGO has notably presented numerous active sites and enhanced contact sites with the electrolyte solution during the faradic reaction. The as-prepared nanocomposite reveals a specific capacity of 223.6 mAh g^−1^ in 1.0 M KOH at 1.0 A g^-1^. Besides, it could retain 89.3% stability after 3000 consecutive galvanostatic charge–discharge cycles at 1.0 A g^−1^ current density. The hybrid supercapacitor, fabricated by activated carbon as an anode site, and NiTe_2_-Co_2_Te_2_@rGO as a cathode site, presents a potential window of 1.60 V with an energy density of 51 Wh kg^−1^ and a power density of 800 W kg^−1^; this electrode is capable of lighting up two red LED lamps and a yellow LED lamp for 20 min, which is connected in parallel. The present work opens new avenues to design and fabrication of nanocomposite electrode materials in the field of supercapacitors.

## Introduction

Energy storage is very important due to the significant increase in human population. Today, fossil fuels are the main energy source closely linked with global warming^1–3^. The presence of high amounts of CO_2_ in atmosphere causes heat on the earth's surface^4^. Evidence from various studies shows that atmospheric CO_2_ has increased by at least 25% since the early nineteenth century. As a result, over the past 150 years, the earth's temperature has risen by more than 1 ^o^F. To survive the earth, using renewable energy is essential to reducing greenhouse gas emissions and air pollution^5^. Therefore, new energy generation technologies like solar^6^, wind^7^, and fuel cells^1^ require devices to store energy.

Batteries^8^, fuel cells^9^, and supercapacitors^10^ are the most important energy storage devices with high energy density and power, optimal life cycle, and mobility. Batteries and supercapacitors are two main electrical energy storage systems developed over the years for portable devices as well as smart grid deployments^11,12^. Supercapacitors can store a large amount of charge compared to conventional capacitors, deliver energy quickly, offer fast charging ability, have a long lifetime, offer superior low-temperature performance, eco-friendly, and have low costs. Moreover, unlike batteries, they do not explode even if it is overcharged.

Electrode materials and electrolytes play an important role in achieving the best performance for commercialization of SCs^13,14^. They affect specific capacitance, operating voltage, energy density, and power density values. Transition metal-based materials have been promising electrode materials for a long time due to their high-rate capability, high capacitance, and low cost. Still, they have limited surface area, low cycling life, and poor electrical conductivity. Hence, (1) controlling the morphology, (2) compositing the electrode materials to generate a synergic effect, (3) doping the element to electrode material to enhance redox reactivity, and (4) defect engineering are several approaches to overcome these deficiencies^15–17^.

Recently transition metal chalcogenides have attracted great attention as promising electrode materials for SCs applications^18,19^. Particularly, transition metal tellurides and selenides, thanks to lower electronegativity and larger atomic radius size than sulfur and oxygen, which lead to advanced chemical, physical and electrochemical properties^20^. The main features of these materials are long cycling life, high electrical conductivity, high mechanical stability, small ionization energy, smooth electron transport, high surface area, enhancing redox-active structures, and high specific capacitance^21–24^. Moreover, the strong covalent bond between tellurium and cobalt/nickel increases the amounts of active sites and electrical catalytic activity. Owing to the atomic orbital overlap of tellurium with cobalt/nickel, the reduced charge-transfer band value results in enhanced supercapacitive property, flexibility, and increased charge transfer^25^.

To date, several electrode materials for supercapacitors based on transition metal chalcogenides have been reported. For example, *Deshagani *et al.^26^ presented the selenide doped of nickel telluride and coated it with poly(N-methylpyrrole) used for SCs application, which shows a specific capacitance of 404 C g^−1^, the energy density of 80 Wh kg^−1^, and power density of 400 W kg^−1^ for asymmetric configuration signifying enhanced synergic effect of the dopant element (Se) on supercapacitor performance. Ye et al. fabricated the 3D hierarchical core–shell structure of NiTe@NiCoSe_2_ as a positive electrode in asymmetric supercapacitors, which exhibited a maximum energy density of 59.8 Wh kg^−1^ and power density of 800 W kg^−1^. This device showed outstanding stability after 10,000 cycles^27^. *Alegaonkar *et al.^28^ presented a facile method for preparing tellurium-rGO for pseudocapacitor application and showed 168.44 F g^−1^ at 1 A g^−1^ in an asymmetric configuration. It has been noted that though those studies show enhanced achievement in the SC field, those materials suffer from uncontrollable experimental conditions and deficient electrode features. Herein, we applied a facile hydrothermal synthesis method to control the morphological and structural features of the as-prepared NiTe_2_-Co_2_Te_2_@rGO nanocomposite. To the best of our knowledge, this is the first study to fabricate NiTe_2_-Co_2_Te_2_@rGO nanocomposite with the hydrothermal method. More importantly, in-depth electrochemical characterization and practical application of SC have been implemented.

In this study, we synthesized a novel spherical NiTe_2_-Co_2_Te_2_ grown on the reduced graphene oxide for a high-performance hybrid supercapacitor. First, CoNi_2_@rGO was synthesized, then Te was introduced in this structure by ion exchange to obtain NiTe_2_-Co_2_Te_2_@rGO, and its morphology changed. The NiTe_2_-Co_2_Te_2_@rGO electrode reveals a specific capacity of 223.6 mAh g^−1^ in a 1.0 M KOH at 1.0 A g^−1^ and still retains 51.10% with the current density increasing to 10 times. Moreover, the NiTe_2_-Co_2_Te_2_@rGO//AC, as a hybrid supercapacitor, delivers a maximal energy density of 51 Wh kg^−1^ at 850 W kg^−1^. The results of this study revealed that NiTe_2_-Co_2_Te_2_@rGO nanocomposite is a promising active material as a hybrid supercapacitor with an acceptable value of energy density *vs.* several related materials.

## Results and discussion

### Structural characterization

**Figure 1:**
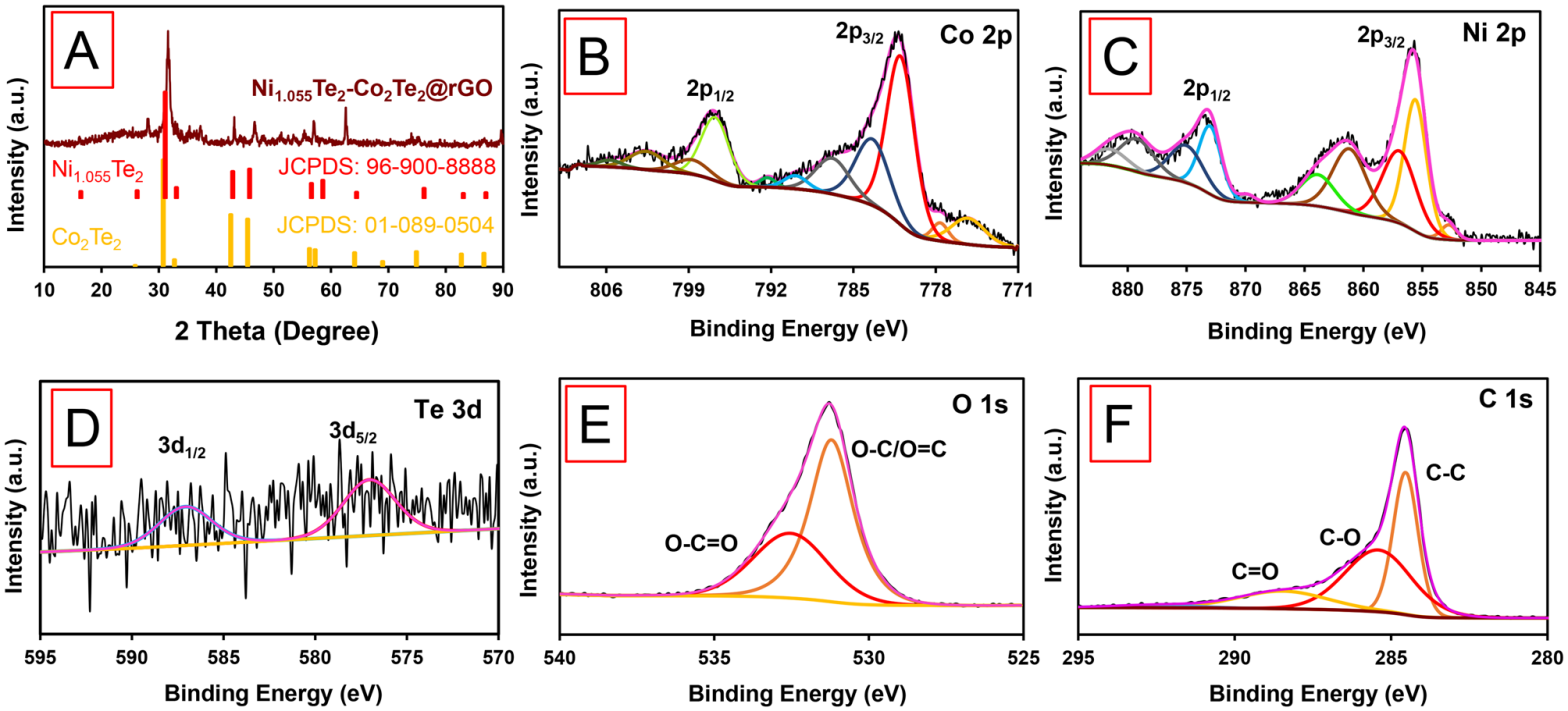
(**A**) XRD spectrum, and (**B**–**F**) High-resolution spectra of Co 2*p*, Ni 2*p*, Te 3*d*, O 1*s*, and C 1*s*, respectively of NiTe_2_-Co_2_Te_2_@rGO.

The X-ray diffraction (XRD) pattern of NiTe_2_-Co_2_Te_2_@rGO (12 h) in Fig. 1A reveals this nanocomposite's crystalline structure and chemical composition. The index peaks are located at 2θ = 16.2 (001), 28.2 (100)/(010), 31.6 (101)/(011), 43.15 (012), 46.75 (110), 57 (021), 62.55 (202), 77.3 (211), and 88.6 (144), corresponding to the peaks of (JCPDS card: 01-089-0504) and (JCPDS card: 96-900-8888)^29^.

The coordinated environment of central metal ions and the valance states were investigated by X-ray photoelectron spectroscopy (XPS). Figure 1B–F proved the structure of NiTe_2_-Co_2_Te_2_@rGO (12 h); two main peaks appeared in the high-resolution spectrum of the Ni 2*p* attributed to Ni 2*p*_3/2_ and Ni 2*p*_1/2_, which indicates the existence of a mixed-valence of (Ni^2+^ and Ni^3+^) and the Co 2*p*_3/2_ and Co 2*p*_1/2_ spectra demonstrated two main peaks contributing to Co^2+^ and Co^3+^ in the nanocomposite. Moreover, the Te 2*d* spectrum reveals two main peaks, Te 2*d*_5/2_ (577.17) and Te 3*d*_3/2_ (586.8), which are attributed to Te^2−^^30^. The O 1*s* peaks at 531.8 and 532.6 eV are associated with the C=O/C–O and water/carboxylic groups, respectively. C 1*s* spectrum shows peaks at 284.5 (C–C bond), 285.6 (C–O bond), and 288.6 (C=O bond) eV in rGO. Consequently, it is detected the valence state and composition of NiTe_2_-Co_2_Te_2_@rGO to investigate the energy storage mechanism.

**Figure 2:**
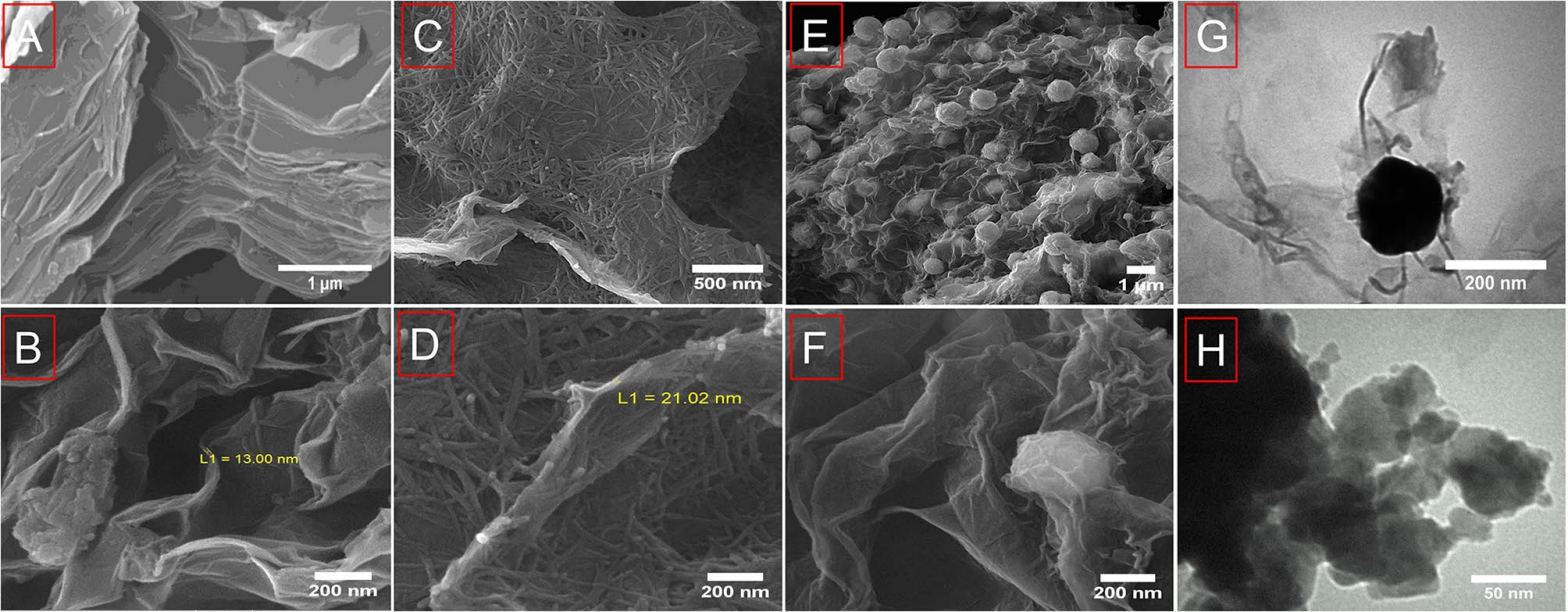
FE-SEM images of (**A**, **B**) GO, (**C**, **D**) CoNi_2_@rGO and (**E**–**F**) NiTe_2_-Co_2_Te_2_@rGO; and TEM images of NiTe_2_-Co_2_Te_2_@rGO (**G, H**)﻿.

The morphology of NiTe_2_-Co_2_Te_2_@rGO (12 h) was investigated through microscopy images. Figure 2A–F illustrate field-emission scanning electron microscopy (FE-SEM) images of (A–B) rGO, (C–D) CoNi_2_@rGO, and (E–F) NiTe_2_-Co_2_Te_2_@rGO. GO image shows dense layers with an effective surface to enhance the rapid diffusion of the ions. CoNi_2_ shows the nanowire morphology on rGO nanosheets with an average thickness of about 25 nm. The spherical NiTe_2_-Co_2_Te_2_@rGO from CoNi_2_ were grown on rGO nanosheets and distributed uniformly. Moreover, this structure may provide an extensive contact area between the electrode and electrolyte because having a large specific surface area. The TEM images (Fig. 2G–H) of NiTe_2_-Co_2_Te_2_@rGO are in good agreement with the FE-SEM images. To identify the composition of the NiTe_2_-Co_2_Te_2_@rGO, the EDX and elemental mapping analysis proved the existences of Ni, Co, Te, O, and C, and the distribution of elements is highly uniform (Fig. S1). Moreover, Fig. S1 exhibits that mentioned elements are uniformly distributed in the entire architecture of this composite.

The electronic structure of GO and NiTe_2_-Co_2_Te_2_@rGO materials was analyzed by Raman spectroscopy as shown in Fig. S2A. In both spectra, the band at 1555 cm^−1^ indicates the tensile vibration of the *sp*^*2*^ hybrid plate, denoted G band, and represents the graphite structure. The band at 1324 cm^−1^ indicates the defect of crystal lattices of *sp*^*3*^ carbon atoms, which is characterized by the D band^31^. The Raman spectra of NiTe_2_-Co_2_Te_2_@rGO displayed a blue shift in G and D-band as compared to the GO spectrum. This may be because of the enhancing number of defects in presence of Te. Another peak appeared at ~ 980 cm^−1^ region in the NiTe_2_-Co_2_Te_2_@rGO due to the formation of metal hydroxide/telluride bonds that confirmed good crystallinity with strong interaction and homogeneous decoration of its particles with rGO sheets. Besides, the I_D_/I_G_ suggests the graphitic degree of carbon materials. The I_D_/I_G_ ratio was calculated as 0.78 and 1.0 for GO and NiTe_2_-Co_2_Te_2_@rGO, respectively which indicated the generation of more in the composite.

The N_2_ adsorption/desorption isotherm curve and the Barrette–Joyner–Halenda (BJH) pore size distribution of NiTe_2_-Co_2_Te_2_@rGO were illustrated in Fig. S2B. Based on the form of the hysteresis loop on the curve, it can be classified as type H3, indicating that pores are slit-like. Moreover, Based on BET and BJH data, the specific surface area, average pore size, and total pore volume are calculated as 34.49 m^2^ g^−1^, 26.6 nm, and 0.23 cm^3^ g^−1^, respectively. According to the results obtained from the reported analysis, it can be concluded that the NiTe_2_-Co_2_Te_2_@rGO is successfully synthesized.

### Supercapacitor characterization

The electrochemical properties of CoNi_2_@rGO, which were synthesized at different times (8, 12, and 16 h), were evaluated by CV and GCD techniques in a three-electrode setup in 1.0 M KOH. Figure S3A displays comparable CV curves of CoNi_2_@rGO at a scan rate of 20 mV s^−1^. Redox peaks that appear in CV curves reveal this electrode's pseudocapacitance nature^32^. As illustrated, CoNi_2_@rGO was synthesized for 12 h, revealing a higher redox peak current density and the integrated area under the CV curve. The second synthesis step for obtained NiTe_2_-Co_2_Te_2_@rGO was performed at various times (8, 12, and 16 h). The CV curves of this structure show the same trend as CoNi_2_@rGO, besides NiTe_2_-Co_2_Te_2_@rGO (12 h) has better reversibility in the same conditions (Fig. S3B). The corresponding GCD curves depict the same result; when synthesized for 12 h, both structures show higher specific capacity at 1 A g^−1^ current density (Fig. S3C,D). Hence, 12 h is the optimal time for both two steps of synthesis due to may be better orbital overlapping and energy band gap decrease.

**Figure 3:**
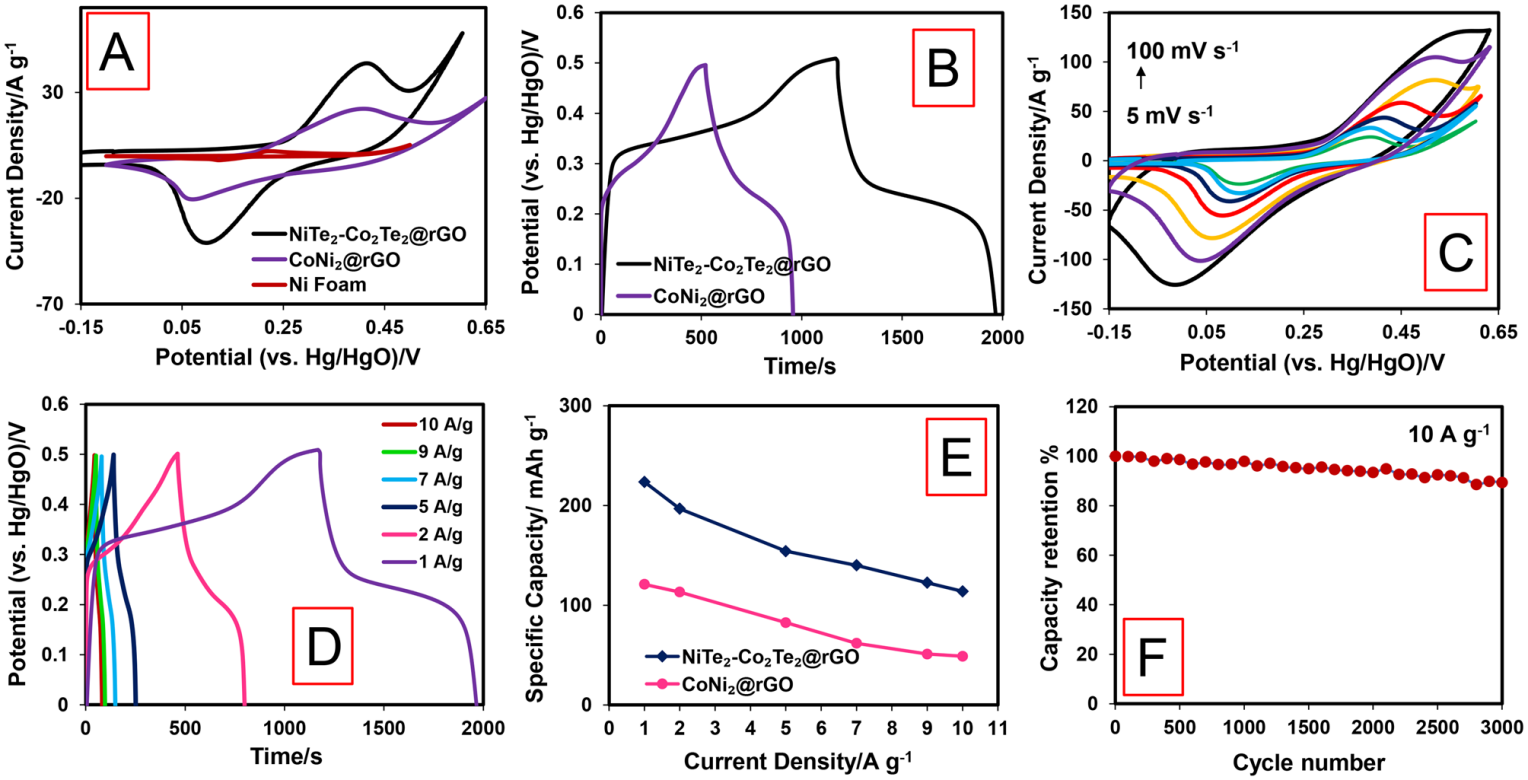
Electrochemical performance measurements in a three-electrode system; (**A**) CV curves of the NF, CoNi_2_@rGO, and NiTe_2_-Co_2_Te_2_@rGO at a scan rate of 20 mV s^−1^, (**B**) GCD curves of the CoNi_2_@rGO and NiTe_2_-Co_2_Te_2_@rGO electrodes at a current density of 1 A g^−1^, **(C)** CV curves of NiTe_2_-Co_2_Te_2_@rGO at different scan rates of 5–100 mV s^−1^, (**D**) GCD curves of the NiTe_2_-Co_2_Te_2_@rGO electrode at various current densities, (**E**) The relevance of the specific capacity of the CoNi_2_@rGO and NiTe_2_-Co_2_Te_2_@rGO electrodes with current densities, and (**F**) Cycling life of NiTe_2_-Co_2_Te_2_@rGO electrode at 10.0 A g^−1^ in 1.0 M KOH.

To investigate the synergic effect between Ni, Co, and Te in tellurides compounds, the CV curves of nickel foam (NF), CoNi_2_@rGO, and NiTe_2_-Co_2_Te_2_@rGO are compared at a scan rate of 20 mV s^−1^ in 1.0 M KOH. The NiTe_2_-Co_2_Te_2_@rGO reveal a larger integrated area under the CV curve and a higher peak current density (Fig. 3A). Furthermore, the corresponding GCD curves are illustrated in (Fig. 3B), and The NiTe_2_-Co_2_Te_2_@rGO reveals the longest discharge time. Hereupon, tellurizing the CoNi_2_@rGO shows the best capacity performance due to enhancing the number of active sites and interconnection between structures and is selected for further investigation.

Figure 3C depicts CV curves of NiTe_2_-Co_2_Te_2_@rGO at different scan rates (5–100 mV s^−1^) in KOH 1.0 M. At lower scan rates, there are two distinct anodic peaks, which are attributed to the Co^2+^/Co^3+^ and Ni^2+^/Ni^3+^ oxidation peaks, but at the higher scan rates, they merged. Moreover, the redox peak of NiTe_2_-Co_2_Te_2_@rGO is illustrated in these curves during the electrochemical process. By augmenting the scan rates, the integrated area under the CV curves tends to be bigger, but the curves' shape is not significantly changed. Thus, the reversible faradic reaction of the NiTe_2_:Co_2_Te_2_@rGO was authenticated, and the rapid ion diffusion is recommended based on previous literature as follows^30^:

1 $${\text{Ni}}/{\text{CoTe }} + {\text{ OH}}^{ - } \to {\text{ Ni}}/{\text{CoTeOH }} + {\text{ e}}^{ - }$$

2 $${\text{Ni}}/{\text{CoTeOH }} + {\text{ OH}}^{ - } \to {\text{ Ni}}/{\text{CoTeO }} + {\text{ H}}_{{2}} {\text{O }} + {\text{ e}}^{ - }$$

Figure 3D displays the GCD curves of NiTe_2_-Co_2_Te_2_@rGO at the different current densities (1–10 A g^−1^). The specific capacity was 223.6, 196.6, 154.2, 140.0, 122.5, and 113.8 mAh g^−1^ at 1, 2, 5, 7, 9, and 10 A g^−1^, respectively. Hence, GCD curves gradually shift left and have a quasi-symmetric shape, revealing great rate performance and coulombic efficiency. The NiTe_2_-Co_2_Te_2_@rGO can keep higher capacity retention of 51.1% than 40.3% of the CoNi_2_@rGO, with the current density augmenting 10 times. Therefore, it diffuses ions faster than CoNi_2_@rGO through the electrolyte during the GCD process (Fig. 3E). Cyclic stability is the essential characteristic parameter for investigating supercapacitor performance. Figure 3F displays the 89.3% initial specific capacity of NiTe_2_-Co_2_Te_2_@rGO maintained after 3000 successive GCDs performed at a 10 A g^−1^ current density in 1.0 M KOH. The result confirms its desirable stability, but a reduction in the specific capacity resulted from the destruction of NCH/PrGO and a loss of its active sites. Table S1 depicts NiTe_2_-Co_2_Te_2_@rGO electrode presents good specific capacity than recently reported related electrode materials.

**Figure 4:**
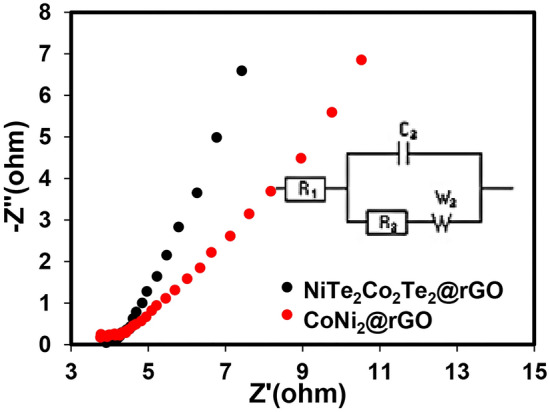
Nyquist plots of the CoNi_2_@rGO and NiTe_2_-Co_2_Te_2_@rGO electrodes from 100 kHz to 100 mHz at open circuit potential (Inset: equivalent circuit).

Electrochemical impedance spectroscopy (EIS) analysis was performed to study the electrode materials' resistive properties. The EIS of NiTe_2_-Co_2_Te_2_@rGO and CoNi_2_@rGO were tested at 100 kHz–100 mHz in 1.0 M KOH (Fig. 4). In the high-frequency region, the semicircle appears to be attributed to the charge transfer process, and the vertical line in the low-frequency region corresponds to the diffusion of the ions at the electrode/electrolyte interfaces. The Nyquist plot of NiTe_2_-Co_2_Te_2_@rGO illustrates a slope close to 90° revealing the fast ions' mobility between the electrode and electrolyte. The NiTe_2_-Co_2_Te_2_@rGO curve was fitted with the Randles circuit model by Z-view software and the corresponding equivalent circuit display inset of Fig. 4. All the parameters to investigate the diffusion of the electrolytes on the electrode's surface, including W_0_ (Warburg element), CPE-P (Constant phase angle exponent), CPE-T (pseudo-capacitance), R_s_ (Solution resistance), and R_ct_ (Charge transfer resistance) are reported in Table 1^33^. W_0_ can be divided into three components: W–R (Ohmic resistance), W–T (diffusion time constant), and W–P (Warburg phase exponent). Accordingly, the NiTe_2_-Co_2_Te_2_@rGO has the lowest charge-transfer resistance due to fast ion diffusion kinetics and electronic conductivity. Moreover, it has the lowest W-R and W-T values illustrating lower shorter ion diffusion and lower Ohmic resistance. According to these electrochemical analyses, the NiTe_2_-Co_2_Te_2_@rGO electrode displays great performance and can be used in supercapacitors.

**Table 1:** The EIS parameters of CoNi_2_@rGO and NiTe_2_-Co_2_Te_2_@rGO.

Electrode	R_s_ (Ω)	R_ct_ (Ω)	CPE-T (s^–n^) × 10^–5^	CPT-P (n)	W–R (Ω)	W–P	W–T (s)
CoNi_2_@rGO	3.76	1.92	68.5	0.91	19.2	0.65	0.042
NiTe_2-_Co_2_Te_2_@rGO	3.77	0.51	9.5	0.84	4.1	0.47	0.11

**Figure 5:**
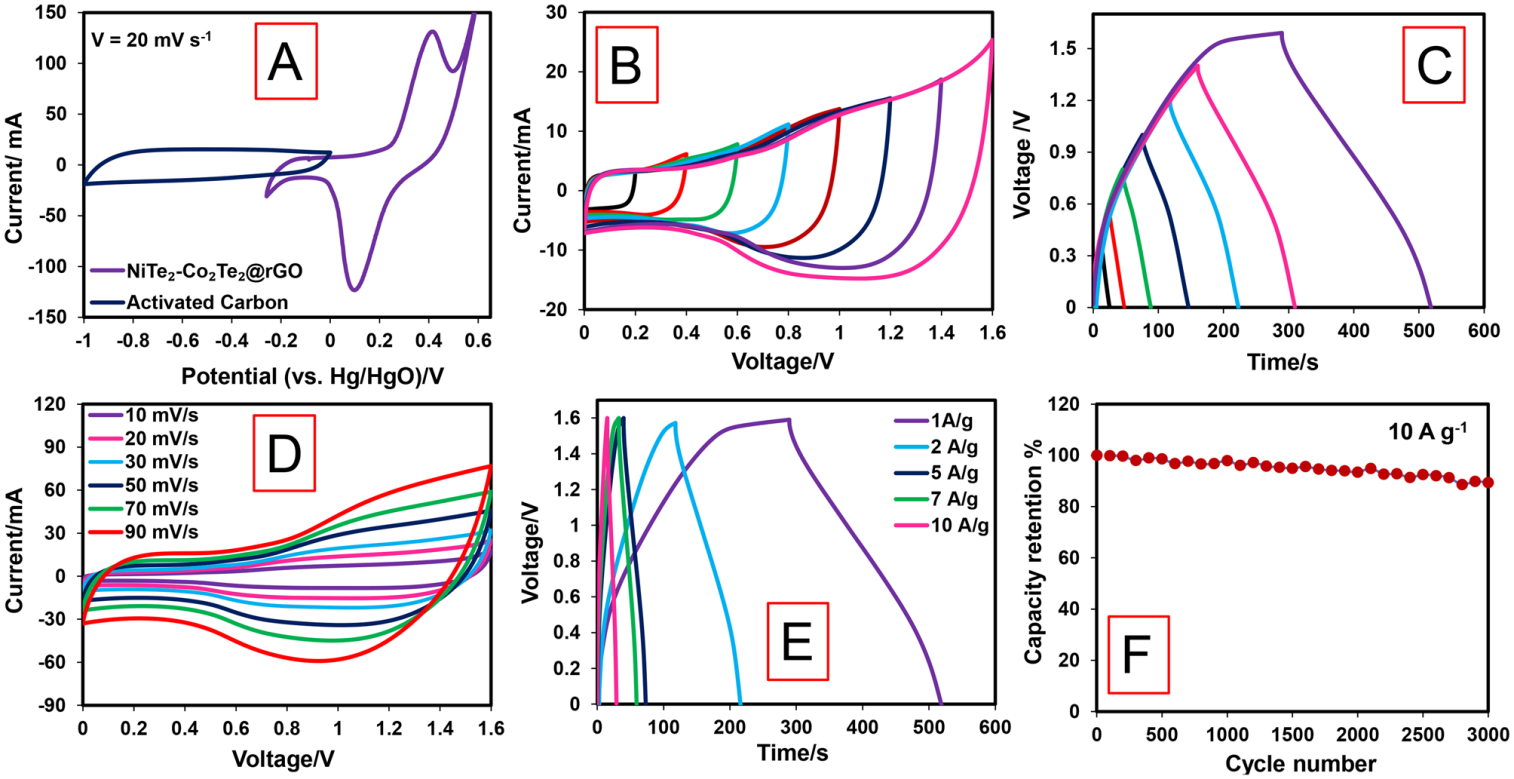
Electrochemical performance measurements in HSC device: (**A**) CV curves of the activated carbon and NiTe_2_-Co_2_Te_2_@rGO electrodes at a scan rate of 20 mV s^−1^ in a three-electrode system in 1.0 M KOH, (**B**) CV curves at different potential windows (0.2–1.6 V) at a scan rate of 20 mV s^−1^, (**C**) GCD curves at different potential windows (0.2–1.6 V) at a current density of 1.0 A g^−1^, (**D**) CV curves at various scan rate (10–90 mV s^−1^), (**E**) GCD curves at different current densities (1–10 A g^−1^), and (**F**) cycling performance at 10 A g^−1^ of the NiTe_2_-Co_2_Te_2_@rGO HSC device.

The activated carbon was utilized as a negative electrode, and NiTe_2_-Co_2_Te_2_@rGO as a positive electrode to fabricate a hybrid supercapacitor (HSC) device with 1.0 M KOH. Figure 5A displays the CV curves of Activated carbon and NiTe_2_-Co_2_Te_2_@rGO in a three-electrode system to evaluate a stable working potential of negative and positive electrodes at scan rates of 20 mV s^−1^. The working voltage of the NiTe_2_-Co_2_Te_2_@rGO//AC HSC device was studied with CV and GCD curves at various voltage windows (Fig. 5B,C). Both CV and GCD curves reveal that the working voltage of this device is 0–1.6 V for further electrochemical measurements. The CVs were performed at different scan rates (10–90 mV s^−1^). The shape of the CV confirmed the capacitive behavior of both electric double-layer capacity and pseudocapacitance (Fig. 5D). The GCD curves of the HSC device were performed at 1 to 10 A g^−1^. The specific capacity was calculated as 64 mAh g^−1^ at 1 A g^−1^, and the retention of NiTe_2_-Co_2_Te_2_@rGO//AC HSC device was ~ 63% (41 F g^−1^) at 10 A g^−1^ (Fig. 5E). Therefore, the high-rate capability reveals the fast kinetics properties of this device.

**Figure 6:**
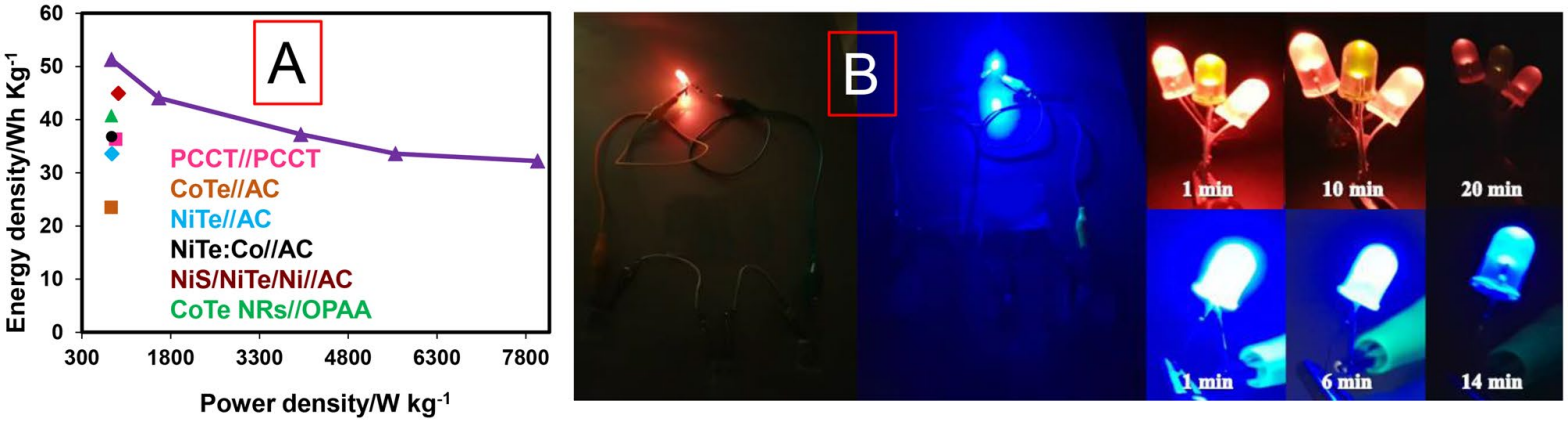
(**A**) Ragone plot of NiTe_2_-Co_2_Te_2_@rGO//AC HSC device vs. other Te-based//AC HSC devices^18,22,34–37^ and (**B**) The yellow and two red LEDs (8 mm) connected in parallel, and a blue LED (8 mm) turned on by three NiTe_2_-Co_2_Te_2_@rGO//AC HSC device.

The stability of NiTe_2_-Co_2_Te_2_@rGO//AC HSC device was tested by consequence GCD cycles at 10 A g^−1^. The specific capacity of NiTe_2_-Co_2_Te_2_@rGO//AC HSC device after 3000 GCD cycles decreases by about ~ 18% of its initial value. As a result of the many harsh redox reactions, the HSC device gradually lost its capacity performance after 3000 cycles (Fig. 5F). The energy density and power density were obtained from GCD curves at different current densities, and the Ragone plot is presented in Fig. 6A. The fabricated HSC device delivered a maximum 51 W h Kg^−1^ energy density at 800 W Kg^−1^ power density. The device also retained the energy density of 32 W h Kg^−1^ when the power density increased to 8000 W Kg^−1^. Besides, the energy density and power density of this device compared with other related HSCs show this device's great performance. Three assembly devices connected in series could be able to light a yellow and two red LEDs (8 mm) in parallel, and a blue LED (8 mm) for 20 and 14 min, respectively (Fig. 6B).

## Methods

### Synthesis of NiTe_2_-Co_2_Te_2_@rG

Firstly, GO was synthesized by the modified hummer method^38^. 100 mg of GO was added to 50 mL DI water and ultrasonicated for 1 h to obtain a uniform suspension (180 kW). Then, the obtained suspension was centrifuged at a speed of 2500 for 15 min, and the supernatant solution was separated. 1.6 mmol Ni(NO_3_)_2_·6H_2_O and 0.8 mmol Co(NO_3_)_2_·6H_2_O were added to the supernatant solution and stirred for 30 min. After that, 10 mmol urea as a reducing agent was added to the suspension, and it was stirred for 45 min and ultrasonicated for 15 min. The final suspension was transferred to the 100 ml Teflon-lined stainless-steel autoclave and kept for 8, 12, and 16 h at 120 °C. Finally, the obtained product (CoNi_2_@rGO) was freeze-drying for 24 h.

**Figure 7:**
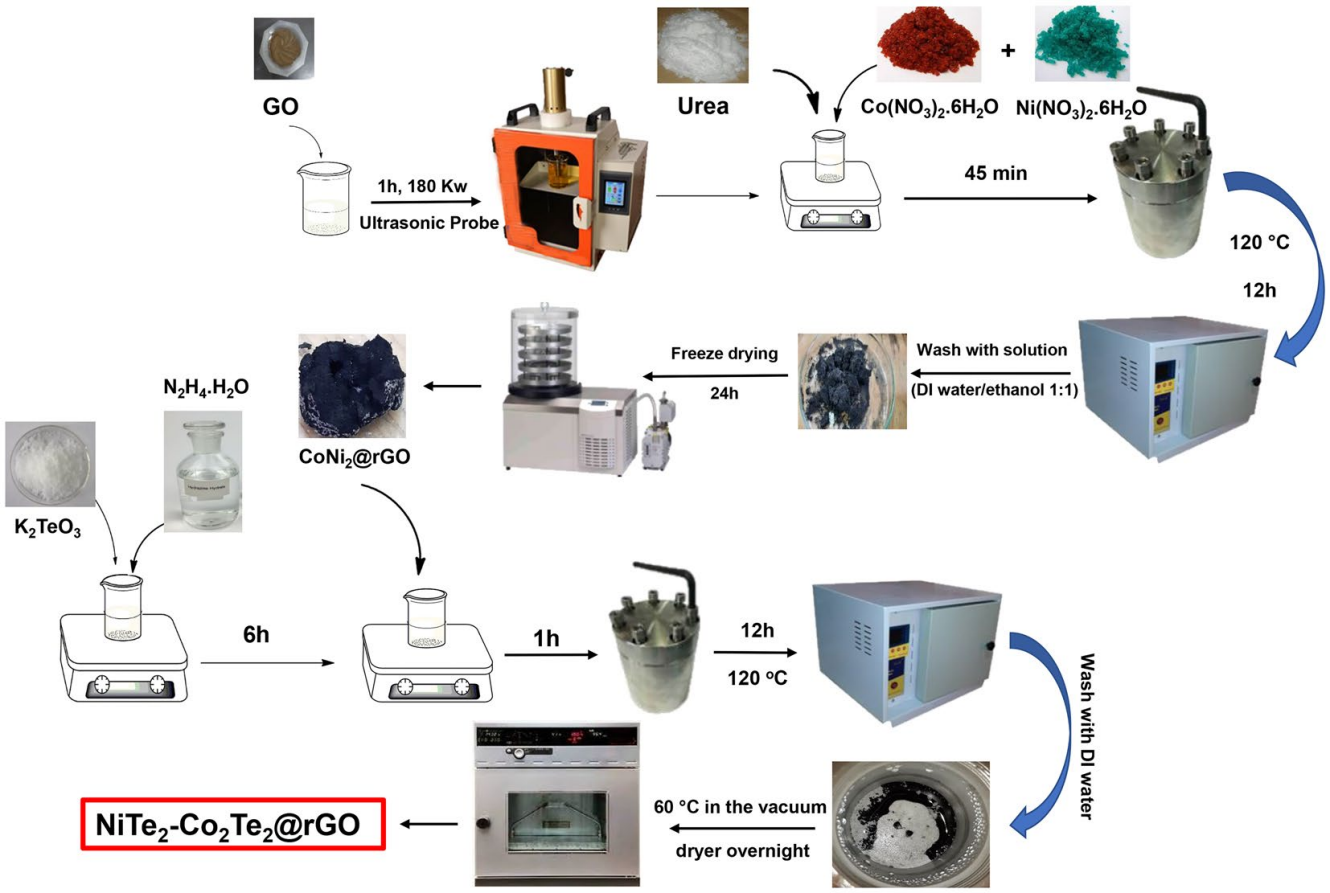
The synthesis procedure of NiTe_2_-Co_2_Te_2_@rGO.

In the next step, 0.7 mmol of K_2_TeO_3_ was dissolved in 60 mL DI water, and 10 mL hydrazine monohydrate as a reducing agent was added, then it was stirred for 6 h. Afterward, 80 mg of CoNi_2_@rGO was added to the obtained solution and stirred for 1 h. This suspension was transferred to the 100 mL Teflon-lined stainless-steel autoclave and kept for 8, 12, and 16 h at 120 °C. The obtained Ni_1.055_Te_2_-Co_2_Te_2_@rGO was dried in a vacuum dryer for 6 h at 60 °C (Fig. 7).

Apparatus, electrochemical measurements, assembling and electrochemical measurements of the hybrid supercapacitors (HSCs) are presented in the Supplementary Information.

## Conclusion

This paper presented the successful approach to synthese spherical NiTe_2_-Co_2_Te_2_ growing on rGO from CoNi_2_ via two-step hydrothermal methods by anion exchange method. The optimum structure of this material for supercapacitor application was chosen by synthesizing both NiTe_2_-Co_2_Te_2_@rGO and CoNi_2_@rGO at different times (6, 12, and 18 h), and 12 h is selected as a reaction time. Various characterization methods, such as XRD, XPS, FE-SEM, EDS, elemental mapping, and TEM were used to reveal the physiochemical properties of NiTe_2_-Co_2_Te_2_@rGO. Moreover, the electrochemical behavior of this electrode was analyzed by CV, GCD, and EIS, illustrating that this structure exposes abundant active sites, and redox reactions can be accelerated, further improving the energy storage effect as well as enhancing the efficiency of energy storage. Besides, the hybrid supercapacitor was assembled on NiTe_2_-Co_2_Te_2_@rGO (as the positive electrode) and AC (as the negative electrode), resulting in a specific capacity of 64 mAh g^−1^ with a high energy density of 51 W h kg^−1^ and power density of 800 W kg^−1^. The excellent electrochemical performance confirms NiTe_2_-Co_2_Te_2_@rGO's potential as an active material for HSCs for future energy storage systems in electronic devices and vehicles. Finally, this work aims to show transition metals telluride's perspective for application in energy storage systems.

## Supplementary Information

Supplementary Information.

## Data Availability

The datasets supporting the conclusions of this article are included within the article.
