# Supplementary material for: Facile synthesis of NiTe2-Co2Te2@rGO nanocomposite for high-performance hybrid supercapacitor
Source: Sci Rep. 2023 Jan 24;13:1364. doi: 10.1038/s41598-023-28581-5 (PMC9873789; doi:10.1038/s41598-023-28581-5)
Supplement: Supplementary file 1 — Supplementary Information. [file 41598_2023_28581_MOESM1_ESM.docx]

**Facile synthesis of NiTe_2_-Co_2_Te_2_@rGO nanocomposite for high-performance hybrid supercapacitor**

Maziar Farshadnia^1^, Ali A. Ensafi ^[[1]](#footnote-1)1,2^, Kimia Zarean Mousaabadi^1^, Behzad Rezaei^1^, Muslum Demir^3^

1. Department of Chemistry, Isfahan University of Technology, Isfahan 84156-83111, IRAN.

2. Adjunct Professor, Department of Chemistry & Biochemistry, University of Arkansas, Fayetteville, AR 72701, USA.

3. Department of Chemical Engineering, Osmaniye Korkut Ata University, Osmaniye, Türkiye.

**Experimental**

**Apparatus**

The chemical structures of the materials were studied by X-ray photoelectron spectroscopy (XPS) (Bes Tec, Germany) and Raman spectroscopy (Teksan Co., Iran). The X-ray diffraction (XRD) patterns were obtained from an AW-XDM300 X-ray diffractometer (Asenware Co., China). The field emission-scanning electron microscope (FE-SEM) images were captured by scanning electron microscopy a MIRA3 (Tescan Co., Czech Republic) with an acceleration voltage at 15 kV equipped with map analysis and energy-dispersive X-ray analysis. Transmission electron microscopic (TEM) analysis was investigated with an Em-208s (Philips Co., Netherlands). Brunauer - Emmet - Teller (BET) surface area analyzer was employed by BELSORP MINI II for surface area and pores size measurements by N2 adsorption-desorption analysis (BEL Co., Japan).

**Electrochemical measurements**

For preparing the working electrode, the 1*1 cm^2^ Nickel foam (NF) as the current collector was immersed in HCl (3 %) and 1:1 (V/V) solution of distilled water and ethanol to clean the surface of its for 5 min, respectively. Then NF was dried in an oven (60°C) for a day. The slurry was prepared by mixing the active material (NiTe_2_-Co_2_Te_2_@rGO), carbon black, and polyvinylidene difluoride (PVDF) (80:15:5 wt%) in N-methyl-2-pyrrolidone (NMP) and was loaded on NF. Afterward, the resulting electrode was dried in an oven (60°C) for a day. The electrochemical measurements of NiTe_2_-Co_2_Te_2_@rGO as an electrode material are studied for supercapacitor application in a three-electrode system, the Ag/AgCl_(3.0M)_ or Hg/HgO_(1.0M NaOH)_ electrode, platinum rod (2 mm diameter), and the prepared electrode was taken as reference electrode, a counter electrode, and working electrode in 1.0 M KOH aqueous solution, respectively.

Assembling and Electrochemical measurements of hybrid supercapacitors (HSCs)

The hybrid supercapacitor (HSC) devices were fabricated using NiTe_2_-Co_2_Te_2_@rGO, activated carbon (AC)/NF, and a piece of *Whatman*® cellulose filter paper as the positive electrode, the negative electrode, and the separator, respectively. According to charge balance theory (q^+^ = q^-^), the optimal mass ratio of positive and negative electrodes (m^+^ /m^-^) was optimized to enhance operating voltage and The ASCs device performance. The mass ratio of solid-state HSCs device is calculated by the equation (1):

(1)

$$\frac{m^{+}}{m^{-}}=\frac{C^{-}{\Delta V}^{-}}{C^{+}{\Delta V}^{+}}$$

*C*^-^ and *C*^+^ represent the specific capacity of AC and NiTe_2_:Co_2_Te_2_@rGO electrodes, respectively. Δ*V*^-^ (V) and Δ*V*^+^ (V) represent the potential window of AC and NiTe_2_:Co_2_Te_2_@rGO electrodes, respectively.

The specific capacity (*Q*) from the discharge curves, the energy density *E* (Wh kg^-1^), and power density *P* (W kg^-1^) of the ASC device were calculated by the equation (2-4):

(2)

$$Q=\frac{I {\Delta t}_{d}}{3.6 m}$$

(3)

$$E=\frac{i \int V dt}{3.6 M}$$

(4)

$$P=\frac{E}{{\Delta t}_{d}} 3600$$

*I* (A) represent the discharge current, *m* (g) represents the total mass of active materials on electrodes, Δ*V* shows operating voltage (V), and Δ*t_d_* (s) represents the discharge time.

All the electrochemical measurements were carried out on a Bio-Logic (SP-300) system, and EC-Lab software was also used to analyze electrochemical curves.


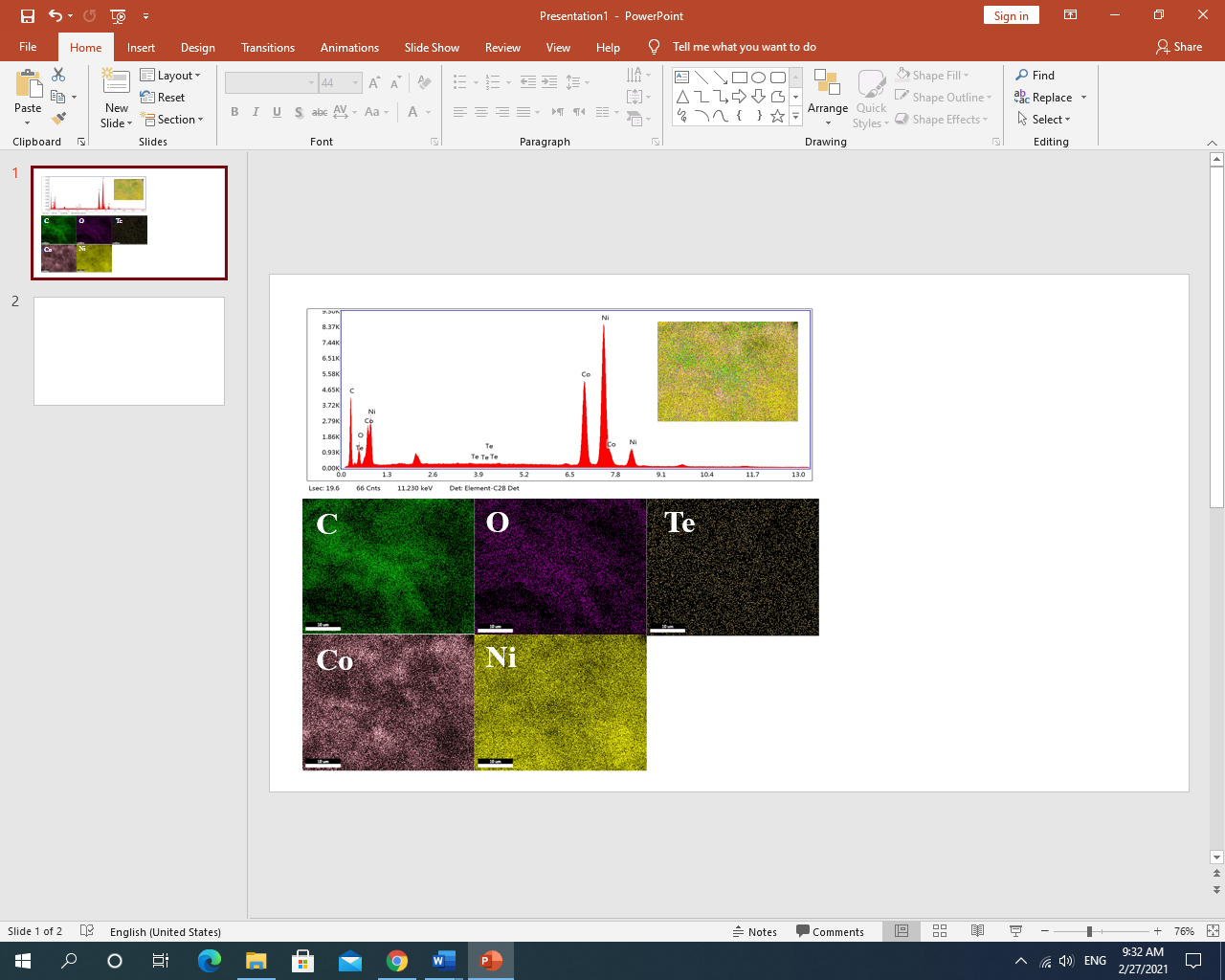


**Figure S1.** ESD spectra and elemental mapping of NiTe_2_-Co_2_Te_2_@rGO.


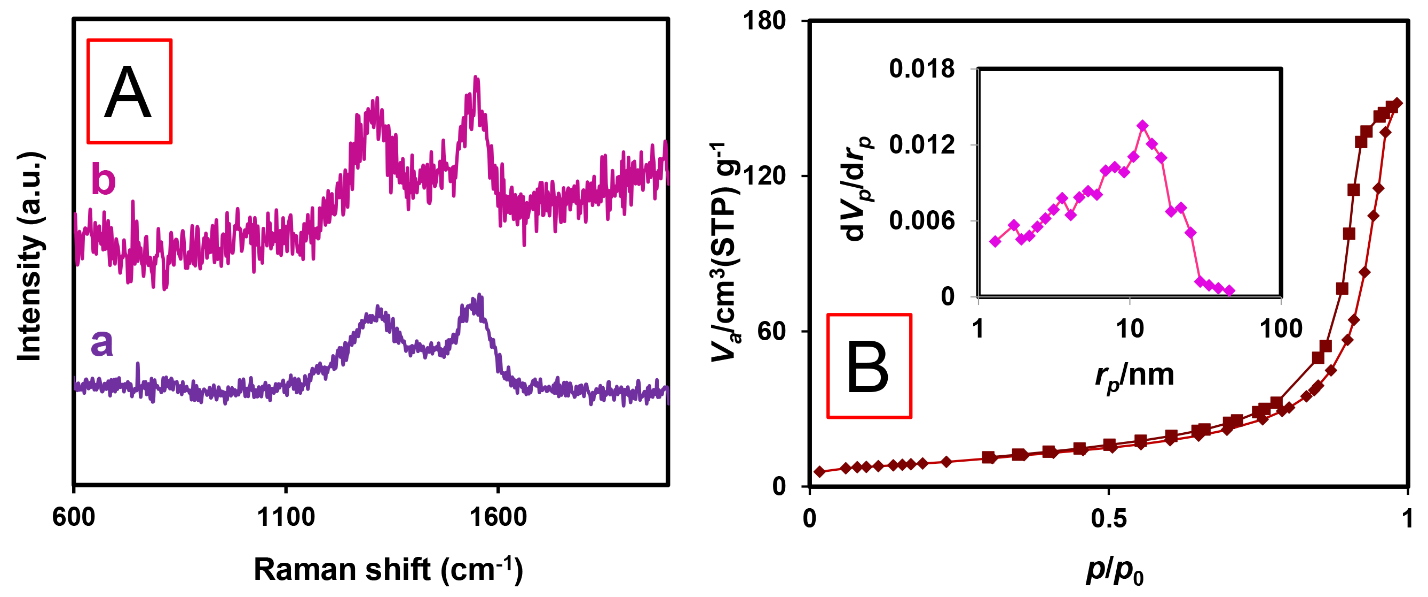


**Figure S2. (A)** The raman spectra of (a) GO and (b) NiTe_2_-Co_2_Te_2_@rGO. **(B)** N_2_ adsorption-desorption isotherm curve of NiTe_2_-Co_2_Te_2_ (Inset: BJH plot).


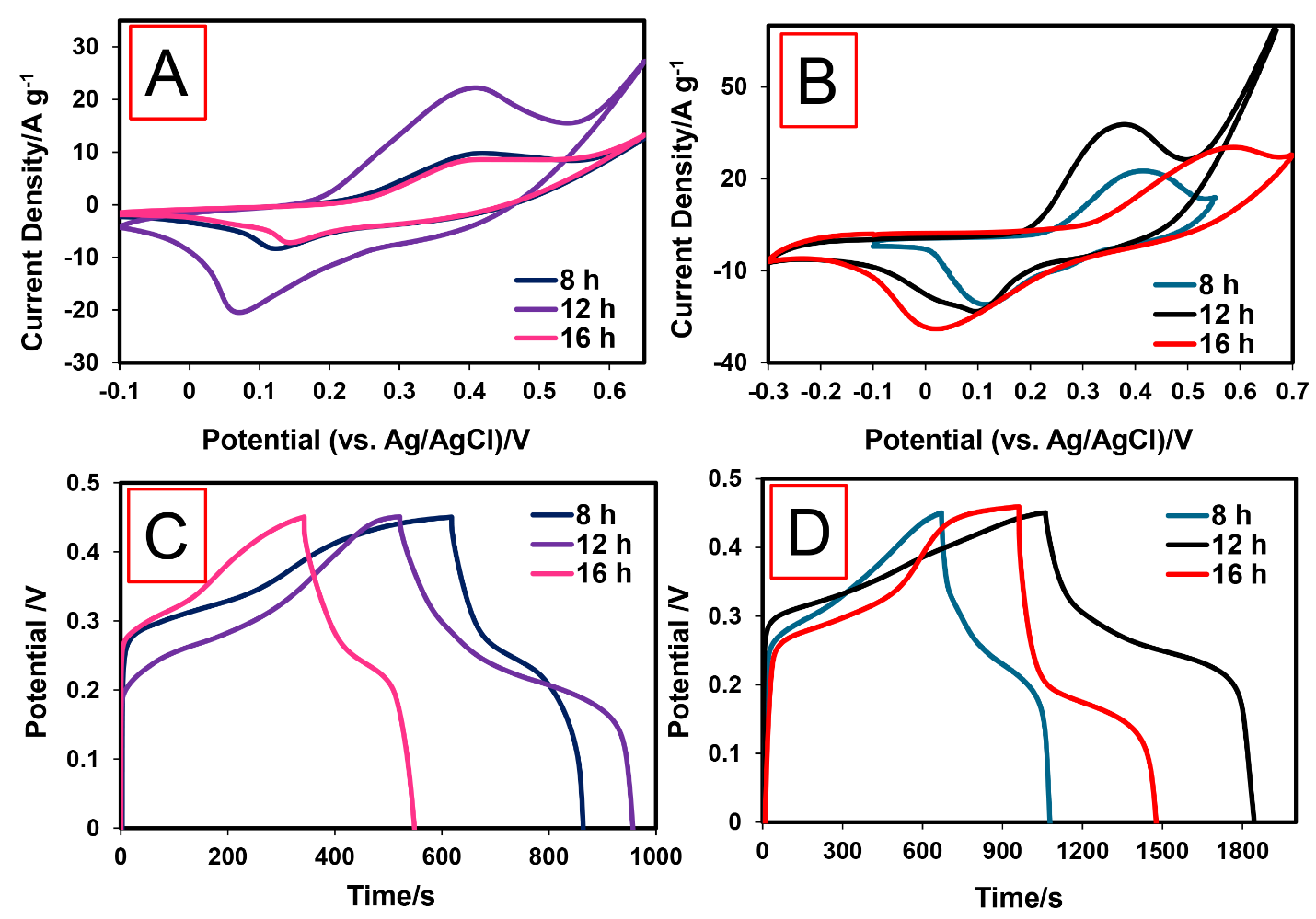


**Figure S3.** Electrochemical performance measurements in a three-electrode system; CV curves of the **(A)** CoNi_2_@rGO, and **(B)** NiTe_2_-Co_2_Te_2_@rGO at a scan rate of 20 mV s^-1^**,** GCD curves of the **(C)** CoNi_2_@rGO and **(D)** NiTe_2_-Co_2_Te_2_@rGO electrodes at a current density of 1 A g^-1^ were synthesized at various times.

**Table S1** A comparison between This work and previous work in Three-electrode system.

| **Active material** | **Media** | **Specific**  **Capacity** | **Current density** |
| --- | --- | --- | --- |
| NiCo_2_Te_4_ ^1^ | KOH | 72 mAh g^-1^ | 2.0 A g^-1^ |
| CF@CoTe_2_-NiTe_2_ ^2^ | KOH | 261.4 mAh g^-1^ | 1.0 A g^-1^ |
| Se-doped NiTe ^3^ | KOH | 998.2 F g^-1^ | 1.0 A g^-1^ |
| NiCo_2_Se_4_/rGO ^4^ | KOH | 1152 F g^-1^ | 2.0 A g^-1^ |
| CoTe ^5^ | KOH | 622.8 F g^−1^ | 1.0 A g^-1^ |
| NiTe_2_-Co_2_Te_2_@rGO  (This study) | KOH | 223.6 mAh g^-1^  1609.9 F g^-1^ | 1.0 A g^-1^ |

**References:**

1. Aparna, M. L., Thomas, T. & Rao, G. R. Battery-like supercapacitive behavior of urchin-shaped NiCo2O4 and comparison with NiCo2X4 (X= S, Se, Te). *J. Electrochem. Soc.* **169**, 20515 (2022).

2. Manikandan, M., Subramani, K., Dhanuskodi, S. & Sathish, M. One-pot hydrothermal synthesis of nickel cobalt telluride nanorods for hybrid energy storage systems. *Energy & Fuels* **35**, 12527–12537 (2021).

3. Ye, B. *et al.* In-situ growth of Se-doped NiTe on nickel foam as positive electrode material for high-performance asymmetric supercapacitor. *Mater. Chem. Phys.* **211**, 389–398 (2018).

4. Ghosh, S., Samanta, P., Samanta, P., Murmu, N. C. & Kuila, T. Investigation of electrochemical charge storage efficiency of NiCo2Se4/RGO composites derived at varied duration and its asymmetric supercapacitor device. *Energy & Fuels* **34**, 13056–13066 (2020).

5. Ye, B. *et al.* Improved performance of a CoTe//AC asymmetric supercapacitor using a redox additive aqueous electrolyte. *RSC Adv.* **8**, 7997–8006 (2018).

1. Corresponding author: Fax: +98-31-33913250, Tel.; +98-31-33913269; E-mail: [Ensafi@iut.ac.ir](mailto:Ensafi@iut.ac.ir); [aensafi@uark.edu,](file:///C:\Users\ensaf\Desktop\Revised\aensafi@uark.edu,) [aaensafi@gmail.com](file:///C:\Users\user\Desktop\aaensafi@gmail.com). [↑](#footnote-ref-1)
